# Di-μ-sulfato-bis­[diaqua­(1*H*-imidazo[4,5-*f*][1,10]phenanthroline)nickel(II)] dihydrate

**DOI:** 10.1107/S1600536808040634

**Published:** 2008-12-10

**Authors:** Wei Zeng, Jin-Hua She, Cui-Juan Wang, Yi Fang

**Affiliations:** a Department of Chemistry and Chemical Engineering, School of Life Science and Bioengineering, SouthWest JiaoTong University, Chengdu, Sichuan 610031, People’s Republic of China; b The Second Research Institute of CAAC, Chengdu, Sichuan 610041, People’s Republic of China

## Abstract

In the title compound, [Ni_2_(SO_4_)_2_(C_13_H_8_N_4_)_2_(H_2_O)_4_]·2H_2_O, the complete dimeric complex is generated by an inversion center. The Ni^II^ atoms are octa­hedrally coordinated by two N atoms from one 1*H*-imidazo[4,5-*f*][1,10]phenanthroline (IP) ligand and two O atoms from two adjacent sulfate ions forming the equatorial plane, with two coordinated water mol­ecules in the axial sites. Both of the sulfate ions act as bidentate-bridging ligands connecting the two Ni^II^ ions, thus generating a binuclear complex. In the crystal structure, O—H⋯O and O—H⋯N hydrogen bonds involving the coordinated and uncoordinated water mol­ecules and N—H⋯O links lead to the formation of a two-dimensional sheet structure developing parallel to (010). Weak π–π stacking inter­actions [centroid–centroid separation = 3.613 (2) Å] between the IP ligands also occur.

## Related literature

For related structures, see: An *et al.* (2007 ▶); Gu *et al.* (2004 ▶). For general background, see: Ross *et al.* (1999 ▶); Xu *et al.* (2003 ▶); Xiong *et al.* (1999 ▶). For details of graph-set theory, see: Bernstein *et al.* (1995 ▶).
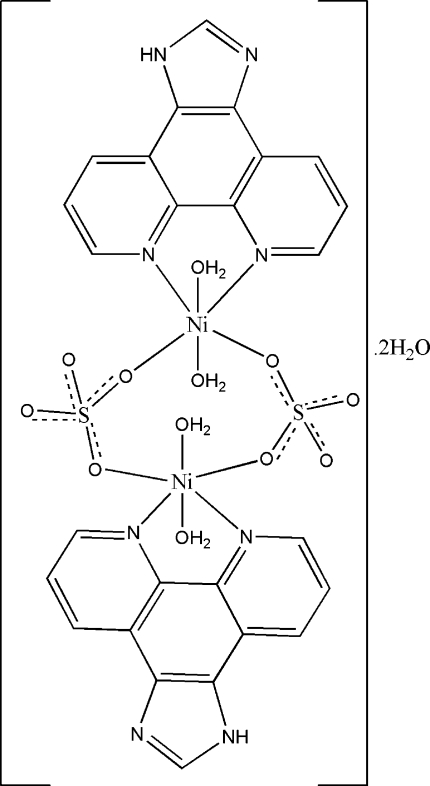


## Experimental

### 

#### Crystal data

[Ni_2_(SO_4_)_2_(C_13_H_8_N_4_)_2_(H_2_O)_4_]·2H_2_O
*M* *_r_* = 858.10
Monoclinic, 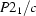

*a* = 10.296 (2) Å
*b* = 9.0560 (18) Å
*c* = 16.836 (3) Å
β = 99.108 (3)°
*V* = 1550.0 (5) Å^3^
*Z* = 2
Mo *K*α radiation
μ = 1.44 mm^−1^
*T* = 298 (2) K
0.28 × 0.20 × 0.13 mm

#### Data collection

Bruker APEXII CCD diffractometer
Absorption correction: multi-scan (*SADABS*; Bruker, 2004 ▶) *T*_min_ = 0.689, *T*_max_ = 0.835
7756 measured reflections
2887 independent reflections
2085 reflections with *I* > 2σ(*I*)
*R*_int_ = 0.040

#### Refinement

*R*[*F* ^2^ > 2σ(*F* ^2^)] = 0.043
*wR*(*F* ^2^) = 0.146
*S* = 0.81
2887 reflections
236 parameters
H-atom parameters constrained
Δρ_max_ = 0.47 e Å^−3^
Δρ_min_ = −0.41 e Å^−3^

### 

Data collection: *APEX2* (Bruker, 2004 ▶); cell refinement: *SAINT* (Bruker, 2004 ▶); data reduction: *SAINT*; program(s) used to solve structure: *SHELXS97* (Sheldrick, 2008 ▶); program(s) used to refine structure: *SHELXL97* (Sheldrick, 2008 ▶); molecular graphics: *ORTEP-3 for Windows* (Farrugia, 1997 ▶); software used to prepare material for publication: *SHELXL97*.

## Supplementary Material

Crystal structure: contains datablocks I, global. DOI: 10.1107/S1600536808040634/hb2874sup1.cif

Structure factors: contains datablocks I. DOI: 10.1107/S1600536808040634/hb2874Isup2.hkl

Additional supplementary materials:  crystallographic information; 3D view; checkCIF report

## Figures and Tables

**Table 1:** Selected bond lengths (Å)

Ni1—O1*W*	2.067 (3)
Ni1—O3^i^	2.095 (3)
Ni1—O1	2.094 (3)
Ni1—N2	2.120 (3)
Ni1—N1	2.136 (3)
Ni1—O2*W*	2.152 (3)

Symmetry code: (i) 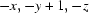.

**Table 2:** Hydrogen-bond geometry (Å, °)

*D*—H⋯*A*	*D*—H	H⋯*A*	*D*⋯*A*	*D*—H⋯*A*
N4—H4*A*⋯O2^ii^	0.86	2.03	2.870 (5)	165
O1*W*—H1*WA*⋯O3*W*^iii^	0.80	1.83	2.630 (4)	180
O1*W*—H1*WB*⋯O4^i^	0.80	1.89	2.695 (4)	180
O1*W*—H1*WB*⋯O3^i^	0.80	2.46	2.873 (4)	114
O2*W*—H2*WA*⋯N3^iv^	0.80	2.00	2.797 (4)	179
O3*W*—H3*WB*⋯O2^i^	0.80	2.00	2.803 (4)	179
O3*W*—H3*WA*⋯O4^v^	0.80	2.04	2.839 (4)	180
O2*W*—H2*WB*⋯O4	0.80	1.96	2.764 (4)	180
O2*W*—H2*WB*⋯O1	0.80	2.50	2.932 (4)	115

Symmetry codes: (i) 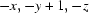; (ii) 
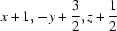
; (iii) 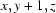; (iv) 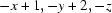; (v) 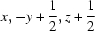.

**Fig. 1.**
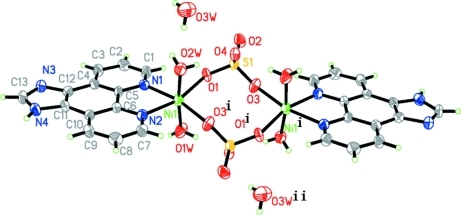
View of (I) with displacement ellipsoids drawn at the 30% probability level. [Symmetry codes: (i)-x,1 - y,-z; (ii) x, y + 1, z]

**Fig. 2.**
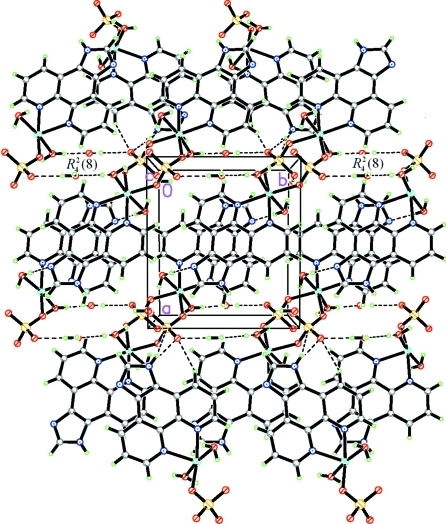
A packing view of the title compound. Hydrogen bonds are shown as dashed lines.

## supplementary crystallographic information

### Comment

Transition-metal complexes with 1,10-phen ligands have shown their employments in catalysis, biochemistry, *etc*. (Ross, *et al.*, 1999; Xu *et al.*, 2003). 1*H*-imidazo[4,5-*f*][1,10]-phenanthroline (IP) is an important derivative of 1,10-phen that has been used to recognize the secondary structure of DNA in Ru(II) complexes (Xiong *et al.*, 1999). Furthermore,the rich electron conjugated rings of IP may be important for providing potential supramolecular recognition sites for π—π aromatic stacking and, *via* the imidazole moiety, IP can form hydrogen-bonding interactions, thus allowing for the formation of supramolecular assemblies. Herein we report the synthesis and characterization of the title compound, (I).

The center of the dimeric complex is located on an inversion center. Each Ni^II^ atom is octahedrally coordinated by two N atoms from one IP ligand and two oxygen atoms from two adjacent sulfate ions forming the equatorial plane, whereas axial positions are occupied by two oxygen atoms of coordinated water molecules (Figure 1). Taking account of these two irregular bond angles [168.06 (11)° for O3—Ni—N1 and 172.06 (12)° for O1W—Ni—O2W], the geometry of copper center is best described as a distorted octahedron (Table 1). The distances of Ni—N and Ni—O bonds are similar to those of related complexes (An *et al.*, 2007; Xu *et al.*, 2003). Both of sulfates taking as bidentated-bridging mode connect Ni^II^ ions, generating a binuclear complex. The separation of Ni—Ni distance is 5.16 (6) Å, which is markedly shorter than the corresponding Ni—Ni distance of 6.132 (4)Å in Ni^II^ analog (Gu *et al.*, 2004). Each IP molecule only binds to Ni^II^ center *via* two nitrogen atoms from two pyridine rings. The IP ligand does not show any abnormal characteristic, with its four bound rings being basically coplanar. One type of π—π stacking interaction between pyridine and imidazole ring from two adjacent IP ligands. The centroid to centroid distances for the further π—π stacking interaction is 3.613 (2)Å [symmetry code = *x*, -*y*, *z* - 1/2], thus indicating weak π—π stacking interaction (Fig. 2).

Intramolecular hydrogen bonds between coordinated water molecules and oxygen atoms from sulfate ions may contribute to its stability (Table 2). Fruthermore, the linking agent is the extensive hydrogen-bonding network involving all the available water molecules and, together with some N atoms of the organic ligand, resulting in the formation of a two-dimensionnal network (Figure 2). For example, the lattice water molecule (O3W) is hydrogen bonded to the O2 and O4 atoms of two related sulfates groups, so generating a *R*^4^_2_(8) motif (Bernstein *et al.*, 1995) (Figure 2).

### Experimental

IP (0.031 g, 0.18 mmol) and NiSO_4_ (0.28 g, 0.11 mmol) were added to acetonitrile (15 ml), the mixture was heated for ten hours under reflux conditions. The resultant solution was then filtered to give a pure solution which was infiltrated by diethyl ether freely in a closed vessel: three weeks later, green blocks of (I) were collected.

### Refinement

All H atoms attached to C atoms, and N atoms were fixed geometrically and treated as riding with C—H distances of 0.93Å (pyridine ring), 0.86 Å (amine group), with *U*_iso_H = 1.2*U*_eq_(C). H atoms of water molecule were located in difference Fourier maps and included in the subsequent refinement using restraints [O—H = 0.82 (1)Å and H···H = 1.38 (2) Å]. In the last stage of refinement, they were treated as riding on their parent O atom with O—H = 0.80Å.

### Crystal data

**Table:**

[Ni_2_(SO_4_)_2_(C_13_H_8_N_4_)_2_(H_2_O)_4_]·2H_2_O	*F*(000) = 880
*M**_r_* = 858.10	*D*_x_ = 1.839 Mg m^−^^3^
Monoclinic, *P*2_1_/*c*	Mo *K*α radiation, λ = 0.71073 Å
Hall symbol: -P 2ybc	Cell parameters from 2887 reflections
*a* = 10.296 (2) Å	θ = 2.5–25.5°
*b* = 9.0560 (18) Å	µ = 1.44 mm^−^^1^
*c* = 16.836 (3) Å	*T* = 298 K
β = 99.108 (3)°	Block, green
*V* = 1550.0 (5) Å^3^	0.28 × 0.20 × 0.13 mm
*Z* = 2	

### Data collection

**Table:**

Bruker APEXII CCD diffractometer	2887 independent reflections
Radiation source: fine-focus sealed tube	2085 reflections with *I* > 2σ(*I*)
graphite	*R*_int_ = 0.040
φ and ω scans	θ_max_ = 25.5°, θ_min_ = 2.5°
Absorption correction: multi-scan (*SADABS*; Bruker, 2004)	*h* = −12→11
*T*_min_ = 0.689, *T*_max_ = 0.835	*k* = −8→10
7756 measured reflections	*l* = −20→20

### Refinement

**Table:**

Refinement on *F*^2^	Primary atom site location: structure-invariant direct methods
Least-squares matrix: full	Secondary atom site location: difference Fourier map
*R*[*F*^2^ > 2σ(*F*^2^)] = 0.043	Hydrogen site location: inferred from neighbouring sites
*wR*(*F*^2^) = 0.146	H-atom parameters constrained
*S* = 0.81	*w* = 1/[σ^2^(*F*_o_^2^) + (0.127*P*)^2^] where *P* = (*F*_o_^2^ + 2*F*_c_^2^)/3
2887 reflections	(Δ/σ)_max_ < 0.001
236 parameters	Δρ_max_ = 0.47 e Å^−^^3^
0 restraints	Δρ_min_ = −0.40 e Å^−^^3^

### Special details

**Table:**

Geometry. All e.s.d.'s (except the e.s.d. in the dihedral angle between two l.s. planes) are estimated using the full covariance matrix. The cell e.s.d.'s are taken into account individually in the estimation of e.s.d.'s in distances, angles and torsion angles; correlations between e.s.d.'s in cell parameters are only used when they are defined by crystal symmetry. An approximate (isotropic) treatment of cell e.s.d.'s is used for estimating e.s.d.'s involving l.s. planes.
Refinement. Refinement of *F*^2^ against ALL reflections. The weighted *R*-factor *wR* and goodness of fit *S* are based on *F*^2^, conventional *R*-factors *R* are based on *F*, with *F* set to zero for negative *F*^2^. The threshold expression of *F*^2^ > σ(*F*^2^) is used only for calculating *R*-factors(gt) *etc*. and is not relevant to the choice of reflections for refinement. *R*-factors based on *F*^2^ are statistically about twice as large as those based on *F*, and *R*- factors based on ALL data will be even larger.

### Fractional atomic coordinates and isotropic or equivalent isotropic displacement parameters (Å^2^)

**Table:**

	*x*	*y*	*z*	*U*_iso_*/*U*_eq_	
Ni1	0.18902 (5)	0.68064 (6)	0.04131 (3)	0.0299 (2)	
S1	−0.03201 (9)	0.60119 (10)	−0.11230 (5)	0.0263 (3)	
N1	0.2570 (3)	0.9021 (4)	0.03289 (18)	0.0266 (7)	
N2	0.3746 (3)	0.6769 (3)	0.11665 (19)	0.0275 (7)	
N3	0.6436 (3)	1.2065 (4)	0.1392 (2)	0.0338 (8)	
N4	0.7470 (3)	1.0131 (4)	0.20327 (19)	0.0340 (8)	
H4A	0.8077	0.9627	0.2321	0.034 (12)*	
O1W	0.0747 (3)	0.7638 (4)	0.12140 (18)	0.0487 (9)	
H1WA	0.0783	0.8400	0.1457	0.058*	
H1WB	0.0340	0.6894	0.1248	0.058*	
O2W	0.2961 (3)	0.5649 (3)	−0.03940 (15)	0.0342 (7)	
H2WA	0.3137	0.6304	−0.0678	0.041*	
H2WB	0.2281	0.5421	−0.0666	0.041*	
O2	−0.0906 (3)	0.6771 (3)	−0.18632 (17)	0.0401 (8)	
O4	0.0613 (3)	0.4873 (3)	−0.13353 (15)	0.0311 (6)	
O1	0.0382 (2)	0.7092 (3)	−0.05661 (16)	0.0300 (6)	
O3W	0.0861 (4)	0.0146 (4)	0.20094 (19)	0.0661 (11)	
H3WB	0.0873	0.1025	0.1963	0.079*	
H3WA	0.0790	0.0137	0.2476	0.079*	
C1	0.1913 (4)	1.0142 (5)	−0.0056 (2)	0.0314 (9)	
H1A	0.1083	0.9961	−0.0347	0.038*	
C2	0.2408 (4)	1.1571 (5)	−0.0042 (2)	0.0339 (10)	
H2A	0.1915	1.2325	−0.0318	0.041*	
C3	0.3637 (4)	1.1857 (4)	0.0385 (2)	0.0308 (9)	
H3A	0.3989	1.2804	0.0395	0.037*	
C4	0.4353 (3)	1.0702 (4)	0.0806 (2)	0.0263 (9)	
C5	0.3759 (3)	0.9290 (4)	0.0770 (2)	0.0231 (8)	
C6	0.4416 (4)	0.8062 (4)	0.1208 (2)	0.0250 (8)	
C7	0.4319 (4)	0.5593 (5)	0.1541 (2)	0.0337 (9)	
H7A	0.3860	0.4705	0.1505	0.040*	
C8	0.5586 (4)	0.5636 (5)	0.1988 (2)	0.0358 (10)	
H8A	0.5960	0.4790	0.2241	0.043*	
C9	0.6267 (4)	0.6942 (4)	0.2048 (2)	0.0323 (10)	
H9A	0.7107	0.6993	0.2347	0.039*	
C10	0.5692 (4)	0.8195 (4)	0.1657 (2)	0.0250 (8)	
C11	0.6261 (4)	0.9622 (5)	0.1659 (2)	0.0300 (9)	
C12	0.5625 (4)	1.0813 (4)	0.1265 (2)	0.0291 (9)	
C13	0.7502 (4)	1.1573 (5)	0.1853 (3)	0.0378 (10)	
H13A	0.8218	1.2175	0.2039	0.045*	
O3	−0.1354 (3)	0.5305 (3)	−0.07522 (17)	0.0352 (7)	

### Atomic displacement parameters (Å^2^)

**Table:**

	*U*^11^	*U*^22^	*U*^33^	*U*^12^	*U*^13^	*U*^23^
Ni1	0.0279 (3)	0.0279 (4)	0.0327 (3)	−0.0050 (2)	0.0010 (2)	−0.0018 (2)
S1	0.0263 (5)	0.0234 (6)	0.0273 (5)	−0.0047 (4)	−0.0016 (4)	0.0013 (4)
N1	0.0259 (17)	0.0269 (19)	0.0260 (16)	−0.0023 (14)	0.0013 (13)	0.0010 (14)
N2	0.0265 (17)	0.0267 (19)	0.0294 (17)	−0.0031 (14)	0.0046 (14)	−0.0015 (14)
N3	0.033 (2)	0.033 (2)	0.0345 (19)	−0.0099 (15)	0.0029 (16)	−0.0019 (15)
N4	0.0277 (18)	0.040 (2)	0.0310 (17)	−0.0034 (16)	−0.0053 (15)	0.0003 (16)
O1W	0.061 (2)	0.0305 (18)	0.061 (2)	−0.0183 (16)	0.0311 (18)	−0.0190 (16)
O2W	0.0315 (15)	0.0345 (17)	0.0370 (15)	−0.0029 (13)	0.0061 (13)	−0.0004 (12)
O2	0.0454 (19)	0.0349 (18)	0.0342 (16)	−0.0055 (13)	−0.0118 (14)	0.0063 (13)
O4	0.0293 (15)	0.0253 (15)	0.0375 (15)	−0.0050 (12)	0.0021 (12)	−0.0043 (12)
O1	0.0253 (14)	0.0252 (15)	0.0348 (15)	−0.0025 (11)	−0.0096 (12)	−0.0042 (11)
O3W	0.126 (4)	0.0292 (19)	0.0479 (19)	−0.015 (2)	0.028 (2)	−0.0107 (16)
C1	0.023 (2)	0.033 (2)	0.036 (2)	−0.0010 (17)	−0.0009 (16)	0.0032 (18)
C2	0.034 (2)	0.031 (2)	0.034 (2)	0.0067 (18)	−0.0012 (18)	0.0047 (18)
C3	0.036 (2)	0.019 (2)	0.036 (2)	−0.0032 (17)	0.0020 (18)	−0.0029 (17)
C4	0.025 (2)	0.028 (2)	0.0251 (18)	−0.0012 (16)	0.0022 (16)	−0.0029 (16)
C5	0.0232 (19)	0.021 (2)	0.0240 (18)	−0.0031 (15)	0.0006 (15)	0.0002 (15)
C6	0.026 (2)	0.025 (2)	0.0239 (19)	−0.0050 (16)	0.0027 (16)	−0.0018 (15)
C7	0.038 (2)	0.027 (2)	0.035 (2)	−0.0067 (18)	0.0029 (18)	0.0014 (18)
C8	0.039 (2)	0.029 (2)	0.038 (2)	0.0085 (19)	0.0021 (19)	0.0065 (18)
C9	0.024 (2)	0.038 (3)	0.032 (2)	−0.0001 (18)	−0.0031 (17)	−0.0009 (18)
C10	0.027 (2)	0.025 (2)	0.0236 (18)	−0.0011 (16)	0.0045 (16)	−0.0009 (15)
C11	0.024 (2)	0.039 (2)	0.0252 (19)	−0.0072 (18)	−0.0023 (16)	−0.0030 (18)
C12	0.031 (2)	0.030 (2)	0.0265 (19)	−0.0052 (17)	0.0061 (16)	−0.0013 (17)
C13	0.038 (3)	0.035 (3)	0.038 (2)	−0.019 (2)	−0.001 (2)	−0.0038 (19)
O3	0.0311 (15)	0.0242 (15)	0.0521 (17)	−0.0034 (12)	0.0116 (13)	0.0031 (13)

### Geometric parameters (Å, °)

**Table:**

Ni1—O1W	2.067 (3)	O3W—H3WB	0.8000
Ni1—O3^i^	2.095 (3)	O3W—H3WA	0.8000
Ni1—O1	2.094 (3)	C1—C2	1.390 (6)
Ni1—N2	2.120 (3)	C1—H1A	0.9300
Ni1—N1	2.136 (3)	C2—C3	1.378 (6)
Ni1—O2W	2.152 (3)	C2—H2A	0.9300
S1—O1	1.464 (3)	C3—C4	1.405 (5)
S1—O3	1.464 (3)	C3—H3A	0.9300
S1—O2	1.467 (3)	C4—C5	1.414 (5)
S1—O4	1.491 (3)	C4—C12	1.415 (5)
N1—C1	1.330 (5)	C5—C6	1.442 (5)
N1—C5	1.349 (5)	C6—C10	1.413 (5)
N2—C7	1.327 (5)	C7—C8	1.400 (6)
N2—C6	1.355 (5)	C7—H7A	0.9300
N3—C13	1.317 (5)	C8—C9	1.371 (6)
N3—C12	1.405 (5)	C8—H8A	0.9300
N4—C13	1.341 (5)	C9—C10	1.396 (5)
N4—C11	1.382 (5)	C9—H9A	0.9300
N4—H4A	0.8600	C10—C11	1.419 (5)
O1W—H1WA	0.8000	C11—C12	1.376 (6)
O1W—H1WB	0.8000	C13—H13A	0.9300
O2W—H2WA	0.8000	O3—Ni1^i^	2.095 (3)
O2W—H2WB	0.8000		
			
O1W—Ni1—O3^i^	87.30 (12)	N1—C1—H1A	118.5
O1W—Ni1—O1	92.33 (12)	C2—C1—H1A	118.5
O3^i^—Ni1—O1	97.62 (11)	C3—C2—C1	119.2 (4)
O1W—Ni1—N2	99.67 (13)	C3—C2—H2A	120.4
O3^i^—Ni1—N2	94.22 (11)	C1—C2—H2A	120.4
O1—Ni1—N2	163.51 (11)	C2—C3—C4	119.2 (4)
O1W—Ni1—N1	85.86 (12)	C2—C3—H3A	120.4
O3^i^—Ni1—N1	168.08 (11)	C4—C3—H3A	120.4
O1—Ni1—N1	92.40 (11)	C3—C4—C5	117.6 (3)
N2—Ni1—N1	77.35 (12)	C3—C4—C12	126.1 (4)
O1W—Ni1—O2W	172.06 (12)	C5—C4—C12	116.4 (3)
O3^i^—Ni1—O2W	84.89 (10)	N1—C5—C4	122.4 (3)
O1—Ni1—O2W	87.31 (11)	N1—C5—C6	117.0 (3)
N2—Ni1—O2W	82.35 (11)	C4—C5—C6	120.7 (3)
N1—Ni1—O2W	102.08 (11)	N2—C6—C10	121.6 (3)
O1—S1—O3	109.72 (16)	N2—C6—C5	116.5 (3)
O1—S1—O2	109.01 (16)	C10—C6—C5	121.9 (3)
O3—S1—O2	109.77 (17)	N2—C7—C8	122.7 (4)
O1—S1—O4	110.14 (15)	N2—C7—H7A	118.7
O3—S1—O4	109.85 (16)	C8—C7—H7A	118.7
O2—S1—O4	108.32 (17)	C9—C8—C7	119.1 (4)
C1—N1—C5	118.5 (3)	C9—C8—H8A	120.5
C1—N1—Ni1	127.0 (3)	C7—C8—H8A	120.5
C5—N1—Ni1	114.3 (2)	C8—C9—C10	119.5 (4)
C7—N2—C6	119.0 (3)	C8—C9—H9A	120.2
C7—N2—Ni1	126.0 (3)	C10—C9—H9A	120.2
C6—N2—Ni1	114.9 (3)	C9—C10—C11	126.5 (4)
C13—N3—C12	103.7 (3)	C9—C10—C6	118.2 (3)
C13—N4—C11	106.0 (3)	C11—C10—C6	115.3 (3)
C13—N4—H4A	127.0	N4—C11—C12	106.5 (4)
C11—N4—H4A	127.0	N4—C11—C10	130.3 (4)
Ni1—O1W—H1WA	131.4	C12—C11—C10	123.2 (3)
Ni1—O1W—H1WB	95.7	C11—C12—N3	109.4 (3)
H1WA—O1W—H1WB	132.3	C11—C12—C4	122.4 (4)
Ni1—O2W—H2WA	101.7	N3—C12—C4	128.1 (4)
Ni1—O2W—H2WB	89.9	N3—C13—N4	114.4 (4)
H2WA—O2W—H2WB	96.3	N3—C13—H13A	122.8
S1—O1—Ni1	130.62 (17)	N4—C13—H13A	122.8
H3WB—O3W—H3WA	96.4	S1—O3—Ni1^i^	139.12 (17)
N1—C1—C2	123.1 (4)		

Symmetry codes: (i) −*x*, −*y*+1, −*z*.

### Hydrogen-bond geometry (Å, °)

**Table:**

*D*—H···*A*	*D*—H	H···*A*	*D*···*A*	*D*—H···*A*
N4—H4A···O2^ii^	0.86	2.03	2.870 (5)	165
O1W—H1WA···O3W^iii^	0.80	1.83	2.630 (4)	180
O1W—H1WB···O4^i^	0.80	1.89	2.695 (4)	180
O1W—H1WB···O3^i^	0.80	2.46	2.873 (4)	114
O2W—H2WA···N3^iv^	0.80	2.00	2.797 (4)	179
O3W—H3WB···O2^i^	0.80	2.00	2.803 (4)	179
O3W—H3WA···O4^v^	0.80	2.04	2.839 (4)	180
O2W—H2WB···O4	0.80	1.96	2.764 (4)	180
O2W—H2WB···O1	0.80	2.50	2.932 (4)	115

Symmetry codes: (ii) *x*+1, −*y*+3/2, *z*+1/2; (iii) *x*, *y*+1, *z*; (i) −*x*, −*y*+1, −*z*; (iv) −*x*+1, −*y*+2, −*z*; (v) *x*, −*y*+1/2, *z*+1/2.
